# Perceived Sociocultural Pressure and Restrained Eating Among Chinese College Students: The Serial Mediating Roles of Self-Objectification and Body-Esteem

**DOI:** 10.3390/nu18132142

**Published:** 2026-07-02

**Authors:** Jingyang Wang, Haoyan Wen, Ximei Chen, Hong Chen

**Affiliations:** 1 Key Laboratory of Cognition and Personality, Southwest University, Ministry of Education, Chongqing 400715, China; 2 Faculty of Psychology, Southwest University, Chongqing 400715, China; 3 Research Center of Psychology and Social Development, Chongqing 400715, China

**Keywords:** restrained eating, tripartite influence model, objectification theory, perceived sociocultural pressure, self-objectification, body-esteem, mental health

## Abstract

Objective: Restrained eating has been recognized as a maladaptive behavior linked to various adverse health outcomes, such as eating disorders and anxiety, which can significantly impact both physical and mental well-being. However, little is known about how perceived sociocultural pressures correlate with restrained eating. Therefore, based on the Tripartite Influence Model and Objectification Theory, we investigated the association between perceived sociocultural pressure and restrained eating as well as the potential mediating role of self-objectification and body-esteem in this association. Method: Participants were 1774 students aged 16–33 years (M_age_ = 19.28 years, 65.80% female) who completed assessments of perceived sociocultural pressure, self-objectification, body-esteem, and restrained eating. Results: The results indicated that after controlling for body mass index and sex, perceived sociocultural pressure was positively correlated with restrained eating. Self-objectification and body-esteem were serial mediators of the association between perceived sociocultural pressure and restrained eating. Discussion: These correlational results suggest that body-esteem may serve as a promising intervention target for programs designed to prevent and reduce restrained eating. In addition, these correlational findings shed light on the prevention and intervention of restrained eating and provide a new theoretical perspective on mental health. In addition, implications and directions for future research were also discussed.

## 1. Introduction

Restrained eating refers to the intentional and continuous restriction of calorie intake to maintain or lose weight, thereby limiting one’s eating behavior and tendencies [1]. This practice is considered maladaptive and associated with a range of negative health consequences, such as eating disorders [2], anxiety [3], reduced body satisfaction and self-esteem, an elevated body mass index (BMI) [4], and negative emotions [5]. It acts as a critical transitional link between dysfunctional body-related cognition and subsequent unhealthy eating behaviors [6]. Notably, restrained eating markedly raises vulnerability to clinical eating pathology [6], which may progress to severe conditions such as anorexia nervosa [7,8] or bulimia nervosa [9,10]. Excessive rumination over weight and physical appearance often triggers body dissatisfaction—individuals may perceive themselves as overweight despite possessing a healthy BMI range—which further reinforces restrained eating patterns and forms a cyclic psychological pathway [11,12]. Furthermore, long-term dietary restriction raises risks of micronutrient deficiency, posing tangible harm to physical development among young populations [13].

In recent years, restrained eating has become prevalent among young Chinese college students [11,14,15]. College students, as a pivotal youth group, are in a critical transition and adaptation stage from adolescence to young adulthood [16]. This period entails heightened responsibility for food choices and serves as a critical window for developing independence and establishing long-term healthy behavioral patterns [17].Therefore, it is important to identify the key influencing factors and mechanisms of restrained eating, which will contribute to the early prevention of unhealthy eating behaviors. Currently, a multitude of scholarly investigations have explored the etiology of restrained eating. Several studies found biological (e.g., BMI [18]), psychological (e.g., self-esteem [19,20,21], self-objectification [22,23]), and sociocultural influences (e.g., weight-related teasing [24], media [14,25]) to predict restrained eating. Given the high prevalence and detrimental health consequences of restrained eating, scholars have continued to explore its antecedents across global contexts [26,27]. Sociocultural pressures regarding appearance (e.g., appearance-based teasing) have emerged as one of the most robust predictors of restrained eating across cultures. However, the nuanced associations between multi-source sociocultural pressure and restrained eating remain inadequately clarified. Thus, it is meaningful to explore sociocultural-related influencing factors linked to restrained eating.

### 1.1. Perceived Sociocultural Pressure and Restrained Eating

The Tripartite Influence Model of sociocultural theory [28,29,30] systematically identifies three core sources of appearance-related sociocultural influence: media, friends, and family. Grounded in this framework, accumulated evidence confirms that perceived sociocultural appearance pressure is closely correlated with restrained eating [31].

Driven by rapid internet development and ubiquitous digital information exposure, public focus on physical appearance has intensified in recent years, accompanied by growing body-related social pressure among young adults. Specifically, media-derived sociocultural pressure, including frequent social media engagement, internalized media pressure [27], repeated selfie browsing [14], and online appearance comparison and body-focused interaction on social platforms [25,32], has been found to correlate with restrained eating.

In parallel, peer-related sociocultural pressure also plays a vital role. Peer relationship dynamics [15], appearance-based teasing [24,33], appearance-related cyberbullying [34], and perceived appearance pressure from friends [35,36] are significantly linked to restrained eating tendencies.

While direct research exploring the association between family appearance pressure and restrained eating remains scarce, the family environment is strongly correlated with individuals’ long-term eating patterns [37,38]. Existing research has verified that parental eating behaviors [39,40] and household structural characteristics [41] are relevant predictors of restrained eating in young people.

To summarize, sociocultural pressures from different sources show significant correlations with restrained eating. However, it remains unclear whether sociocultural pressures originating from distinct sources exert differential correlational strengths on restrained eating. Therefore, the present study will examine the association between sociocultural pressures and restrained eating by analyzing each dimension of sociocultural pressure separately. We propose Hypothesis 1: perceived sociocultural pressure is positively correlated with restrained eating.

### 1.2. The Mediating Role of Self-Objectification

Self-objectification involves perceiving and conceptualizing one’s body from the perspective of an external observer, wherein individuals assess themselves based on their appearance [42]. Individuals with high levels of self-objectification tend to engage in weight-control behaviors including restrained eating [43]. Self-objectification is pervasive among college students and shows close correlational links with restrained eating as a proximal variable [2,23,44]. Furthermore, perceived sociocultural appearance pressure is significantly associated with self-objectification [45]. Specifically, external sociocultural pressure facilitates the internalization of societal thin ideals, which correlates with elevated self-objectification tendencies [46]. A systematic review further indicated that maternal influences, peer interactions, media exposure, and pubertal development can all predict self-objectification experiences [47]. Rooted in Objectification Theory, existing frameworks demonstrate that sociocultural and psychological factors collectively correlate with restrained eating [48]. Accordingly, grounded in Objectification Theory and prior empirical results, we propose Hypothesis 2: self-objectification mediates the association between perceived sociocultural pressure and restrained eating.

### 1.3. The Mediating Role of Body-Esteem

Body-esteem reflects individuals’ positive subjective evaluation of multiple physical attributes of their body [49,50,51]. Cumulative research has confirmed significant correlations among weight, body-esteem, and restrained eating [20,52,53,54,55], and low body-esteem is closely associated with restrained eating [12]. This construct consists of three subdimensions: weight-esteem, appearance-esteem, and attribution-esteem. Among these dimensions, weight-esteem serves as a powerful mediator in pathways relevant to restrained eating [56], and reduced weight-esteem can mediate the association between excessive social media engagement and restrained eating [57]. In addition, consistent associations have been documented between perceived sociocultural pressure and body-esteem. Prior research has validated the mediating role of weight-esteem in the linkage between sociocultural pressure and disordered eating tendencies [58], and elevated perceived thin-ideal sociocultural pressure has been found to correlate with diminished body-esteem [59]. In light of existing empirical evidence, we propose Hypothesis 3: body-esteem mediates the association between perceived sociocultural pressure and restrained eating.

### 1.4. The Serial Mediating Role of Self-Objectification and Body-Esteem

Prospective research has confirmed that self-objectification is significantly correlated with reduced body-esteem [60]. From the perspective of Objectification Theory, external appearance pressure first prompts individuals to conduct outward appearance monitoring, manifested as increased self-objectification; this sustained appearance-centered evaluation further relates to the decline of positive physical self-assessment (body-esteem), which theoretically supports the rationale for selecting the two variables as sequential mediators instead of parallel mediators. In particular, appearance pressures from parents, friends, and the media (e.g., the pressure to be thin) align with mainstream appearance ideals (e.g., thin is beautiful), which correlate with elevated individual self-objectification, body dissatisfaction, and weight concern, sequentially associating with lower body-esteem and weight-esteem. Further, self-objectification is closely correlated with body-esteem and weight concerns [61], indicating that self-objectification and body-esteem may be closely related. Based on this, we further propose Hypothesis 4: self-objectification and body-esteem play a role in the serial statistical mediation of the relationship between perceived sociocultural pressure and restrained eating.

### 1.5. This Study

This study aims to address existing research gaps regarding the association between perceived sociocultural pressure and restrained eating. By integrating the Tripartite Influence Model and Objectification Theory, the current research explores the underlying correlational pathways toward restrained eating, so as to offer theoretical support for future practical intervention design. Based on a large sample of Chinese college students (N = 1774), we examine the correlation between perceived sociocultural pressure and restrained eating, alongside the serial mediating roles of self-objectification and body-esteem within this pathway.

**Figure 1:**
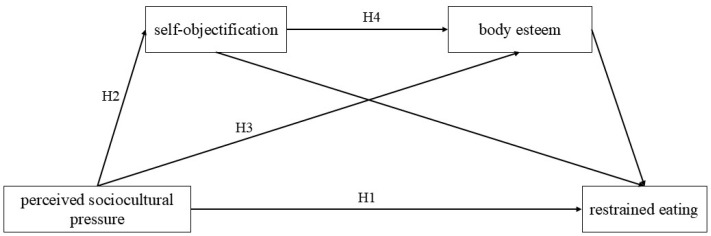
The serial mediation model. This figure exhibits the relationship among perceived sociocultural pressure, self-objectification, body-esteem and restrained eating.

This study further distinguishes distinct dimensions of sociocultural pressure correlated with restrained eating and identifies variables correlated with restrained eating to support the development of targeted prevention strategies and intervention programs. The hypothesized serial mediation model based on the total score of perceived sociocultural pressure is displayed in Figure 1. Subsequent supplementary analyses will further examine the correlational effects of its four subdimensions separately.

Furthermore, abundant empirical evidence reveals prominent gender disparities in body image responses to sociocultural appearance pressure among young adults [62,63]. Females tend to internalize thin ideals from social media more readily and experience higher levels of self-objectification, while males are shaped by unique masculine appearance norms. Accordingly, we carry out exploratory multi-group serial mediation analyses to test gender heterogeneity in subsequent statistical tests.

We adopted a serial multiple mediator model rather than a parallel multiple mediator model based on integrated theoretical considerations. The Tripartite Influence Model defines external multi-source sociocultural triggers including media, friends, and family appearance pressure, whereas Objectification Theory explains the internal cognitive progression from external appearance monitoring to subjective physical self-evaluation. This integrated external–internal pathway is consistent with the biopsychosocial framework of body image proposed by Rodgers et al. [64], which jointly justifies the sequential order of self-objectification and body-esteem as serial mediators rather than parallel mediators. The Tripartite Influence Model and Objectification Theory jointly suggest a sequential associative process in which sociocultural pressure correlates with elevated appearance monitoring (self-objectification), which is further associated with lower body-esteem, and ultimately links to restrained eating. This theorized sequential ordering (sociocultural pressure → self-objectification → body-esteem → restrained eating) aligns conceptually with a serial mediation specification. Although we acknowledge that alternative variable orderings and competing models are statistically plausible, our primary model was determined by this theoretical framework rather than data-driven model selection.

Research Hypotheses

**H1:**  *Perceived sociocultural pressure is positively correlated with restrained eating.*

**H2:**  *Self-objectification acts as a statistical mediator between sociocultural pressure and restrained eating.*

**H3:**  *Body-esteem acts as a statistical mediator between sociocultural pressure and restrained eating.*

**H4:**  *Self-objectification and body-esteem sequentially serve as serial statistical mediators linking perceived sociocultural pressure to restrained eating.*

## 2. Methods

### 2.1. Participants

Data were collected from our ongoing project named the Behavioral Brain Research Project of Chinese Personality. Prior to engaging in data collection processes, such as the measurement of behavioral variables, participants were assessed for self-reported medical conditions, neurological issues, and mental health disorders using a series of binary indicators that reflected the presence or absence of specific ailments and the consumption of psychoactive drugs. Subsequently, participants were encouraged to answer all queries with utmost honesty to reduce the impact of social desirability bias. Ethical approval was granted by the Ethics Committee of the university where the study was conducted. The approval date of the ethical statement is 7 September 2019 (project identification code: SWUPSY19090201). In the preliminary phase of this study, 1816 questionnaires were collected between October 2019 and January 2021. After deleting blank answers and irregular responses, 1774 questionnaires were deemed valid, representing a response rate of 97.69%. The demographic distribution of the final sample comprised 1195 female and 579 male participants. Participants were aged 16 to 33 years (mean [M] = 19.28 years, standard deviation [SD] = 1.10 years) with a BMI (calculated as weight in kilograms divided by height in meters squared; BMI = kg/m^2^) between 14.29 and 35.69 (M = 21.51, SD = 3.12). According to standard BMI classifications, the sample comprised 14.4% underweight (BMI ≤ 18.5), 72.9% normal weight (BMI = 18.6–24.9), 10.8% overweight (BMI = 25–29.9), and 1.8% obese (BMI ≥ 30) individuals.

### 2.2. Measures

#### 2.2.1. Perceived Sociocultural Pressure

The Chinese version of the Perceived Sociocultural Pressure Scale (PSPS) [3,46,65,66] was developed based on the well-established SATAQ-4R theoretical framework proposed by Schaefer et al. [67]. This scale comprises eight items, rated from 1 = almost never to 5 = almost always. The PSPS items can be separated into four subscales measuring the four facets of perceived sociocultural pressure: (a) family (two items; e.g., “I have felt pressure from my family to change my physical appearance”), (b) friends (two items; e.g., “I have felt pressure from my friends to change my physical appearance”), (c) generalized others (two items; e.g., “I have felt a strong message from people that if I want to date or have a date, I have to have a certain physical appearance”), and (d) media (two items; e.g., “I have felt strong messages from the media (e.g., television, magazines) about the need to have a certain physical appearance”). Scores were analyzed by calculating the total scores for the subscales and the total scale. In this study, Cronbach’s alphas were 0.91 for the total scale and 0.91, 0.87, 0.91, and 0.95 for the subscales, respectively, indicating excellent internal consistency.

#### 2.2.2. Restrained Eating

The Chinese version of the Restraint Scale (RS) revised by Kong and colleagues [68] comprises 10 items across two subscales: concern for dieting (six items; sample item: “How often are you dieting?”) and weight fluctuation (four items; sample item: “In a typical week, how much does your weight fluctuate?”). In the RS, Items 5–8 adopt a 4-point Likert response format ranging from 0 (never) to 3 (always), while Items 1–4 and 9–10 use a 5-point scale. Higher total scores represent greater levels of restrained eating. In this study, the Cronbach’s alpha for the scale was 0.76, indicating acceptable internal consistency.

#### 2.2.3. Body-Esteem

The Body-Esteem Scale for Adolescents and Adults (BESAA) [69] is a 23-item tool designed to assess individuals’ cognitive, emotional, and behavioral responses to their body image, providing insights into their self-perception and body-esteem. The BESAA has three subscales: BE-Appearance, BE-weight, and BE-Attribution. In this study, we used only BE-weight to estimate weight-related self-esteem. Using a five-point Likert scale ranging from 0 (never) to 4 (always), participants indicated the degree to which they agreed with each statement (e.g., “I am satisfied with my weight”). Higher scores indicate higher body-esteem. In this study, the Cronbach’s alpha for the scale was 0.89, indicating very good internal consistency.

#### 2.2.4. Self-Objectification

The self-objectification questionnaire [70] was used. The questionnaire required participants to rate a set of 10 attributes that contribute to their body self-concept on a scale of perceived influence. Each attribute was assigned a numerical value corresponding to its relative impact, with nine indicating the highest degree of influence and zero reflecting the least. The attributes under consideration were categorized into two domains: five pertained to physical appearance (e.g., skin), whereas the remaining five were associated with ability (e.g., power). The final score is calculated by subtracting the total score of the five ability-related items from the total score of appearance-related items. Total scores range from −25 to 25. Higher scores represent a higher level of self-objectification. This scale has been effectively used with Chinese participants [32,71].

### 2.3. Data Analysis

Descriptive and correlation analyses and common method bias testing were performed using SPSS 26.0 (IBM Corporation, Armonk, NY, USA). Pearson’s correlation analysis was conducted to examine the associations among perceived sociocultural pressure, self-objectification, body-esteem, and restrained eating. Path analysis was performed using Hayes’ PROCESS Macro v4.2 for SPSS (Andrew F. Hayes, Columbus, OH, USA), which uses bootstrapping and involves repeated sampling from the dataset with replacement (in this case, 5000 bootstrap resamples) to create an approximation of the sampling distribution of the indirect effect and generate confidence intervals for these effects. An indirect effect was considered significant if the confidence interval did not cover zero. Bootstrapping of confidence intervals (CIs) was used to examine the significance of the indirect effects of perceived sociocultural pressure on restrained eating. *p* values, 95% CIs, unstandardized regression coefficients (b), and corresponding standardized coefficients (β) are reported. The advantage of the serial mediation model is that, whereas parallel mediation models assume that no mediator exerts predictive influences another, no such assumption is made in serial mediation. Thus, we can test a specific theoretical sequence among the variables [72].

## 3. Results

### 3.1. Common Method Biases Test

Common method bias is a potential problem when using self-reported measurement methods. The Harman single-factor method was used to check and test for common method bias. There were six factors that presented eigenvalues greater than 1, and the interpretation rate of the first common factor was 29.88%, which was less than 40% [73], indicating no common method bias in the questionnaires used in this study.

### 3.2. Descriptive Statistics and Correlation Analysis

Means, SDs, and bivariate correlations were used to examine the associations among all variables. Descriptive statistics and correlation analyses were conducted for perceived sociocultural pressure, body-esteem, self-objectification, and restrained eating. The results (Table 1) showed that the total score of perceived sociocultural pressure (and its four dimensions), body-esteem, self-objectification, and restrained eating were significantly correlated (all *p* < 0.05). Specifically, the total scores of perceived sociocultural pressure and its four dimensions were significantly positively correlated with self-objectification and restrained eating, whereas body-esteem was significantly negatively correlated with perceived sociocultural pressure and its four dimensions. Additionally, there were significant correlations among sex, BMI, and several other variables. Therefore, sex and BMI were controlled for in subsequent analyses.

### 3.3. Serial-Mediation Analyses

Model 6 of the SPSS PROCESS macro program was used to test the serial mediation effects. Sex and BMI were covariates, total perceived sociocultural pressure and its four dimensions were independent variables, restrained eating was the dependent variable, and body-esteem and self-objectification were the mediating variables. A significance test of the regression coefficient was performed using the bootstrap method (repeated sampling 5000 times) to obtain the estimated value of the parameter and a 95% confidence interval. The analysis results were as follows:

#### 3.3.1. Perceived Sociocultural Pressure and Restrained Eating

Table 2 shows the results of the serial mediation regression analysis. After controlling for sex and BMI, perceived sociocultural pressure was positively associated with self-objectification, *β* = 0.187, *p* < 0.001, *R*^2^ = 0.062, *p* < 0.001. In the second step, with sex, BMI, and self-objectification controlled, perceived sociocultural pressure was negatively associated with body-esteem, *β* = −0.366, *p* < 0.001, *R*^2^ = 0.337, *p* < 0.001; self-objectification also showed a negative association, *β* = −0.074, *p* < 0.001. In the final step, when all variables were included, perceived sociocultural pressure was positively associated with restrained eating, *β* = 0.121, *p* < 0.001. Self-objectification showed a positive association with restrained eating, *β* = 0.059, *p* < 0.01, and body-esteem showed a negative association, *β* = −0.328, *p* < 0.001. The full model accounted for 37.3% of the variance in restrained eating, *R*^2^ = 0.373, *p* < 0.001. The standardized path coefficient plot of the model is provided as Figure S1 in the Supplementary Materials.

The total effect (*B* = 0.202, *β* = 0.256) remained significant after including mediators, with a reduced direct effect (*B* = 0.095, *β* = 0.121). The total indirect effect was 0.107 (95% *CI* [0.089, 0.126]), accounting for 52.90% of the total effect. The decomposition of this total indirect effect into specific paths and their respective percentages is shown in Table 3. The largest indirect contribution was carried by body-esteem (46.90%), whereas the serial pathway through self-objectification and body-esteem was minimal (1.80%). Regarding effect sizes, the completely standardized indirect effect for the body-esteem path fell in the medium range (*β* = 0.120), while the standardized effects for the self-objectification path (*β* = 0.011) and the serial path (*β* = 0.005) were small.

#### 3.3.2. Friends of Perceived Sociocultural Pressure and Restrained Eating

Table 4 presents the serial mediation regression results for friends’ sociocultural pressure. After controlling for sex and BMI, friends of perceived sociocultural pressure was positively associated with self-objectification, *β* = 0.132, *p* < 0.001, *R*^2^ = 0.046. In the second step, with additional controls for self-objectification, friends’ pressure was negatively associated with body-esteem, *β* = −0.293, *p* < 0.001; self-objectification also showed a negative association, *β* = −0.103, *p* < 0.001, *R*^2^ = 0.296. In the final step, when all variables were included, friends’ pressure was positively associated with restrained eating, *β* = 0.089, *p* < 0.001; self-objectification was positively associated, *β* = 0.067, *p* < 0.001, and body-esteem was negatively associated, *β* = −0.347, *p* < 0.001. The full model accounted for 37.0% of the variance in restrained eating, *R*^2^ = 0.370. The standardized path coefficient plot of the model is provided as Figure S2 in the Supplementary Materials.

Table 5 shows the total, direct, and indirect effects. The total effect was *B* = 0.568 (*β* = 0.204), and the direct effect was reduced to *B* = 0.248 (*β* = 0.089) after including the mediators. The total indirect effect was 0.321 (95% *CI* [0.265, 0.383]), accounting for 56.4% of the total effect. Detailed decomposition of the indirect effect into specific paths and their corresponding percentages is provided in Table 5. The largest indirect association was carried by body-esteem (49.8%), whereas the serial path through self-objectification and body-esteem was small (2.3%). The completely standardized indirect effect for the body-esteem path was in the medium range (*β* = 0.102), while effects for the self-objectification path (*β* = 0.009) and the serial path (*β* = 0.005) were small.

#### 3.3.3. Family of Perceived Sociocultural Pressure and Restrained Eating

Table 6 presents the serial mediation regression results for family of perceived sociocultural pressure. After controlling for sex and BMI, family of perceived sociocultural pressure was positively associated with self-objectification, *β* = 0.111, *p* < 0.001, *R*^2^ = 0.041. In the second step, with additional controls for self-objectification, family of perceived sociocultural pressure was negatively associated with body-esteem, *β* = −0.256, *p* < 0.001; self-objectification also showed a negative association, *β* = −0.113, *p* < 0.001, *R*^2^ = 0.275. In the final step, when all variables were included, family of perceived sociocultural pressure was positively associated with restrained eating, *β* = 0.046, *p* < 0.05; self-objectification was positively associated, *β* = 0.071, *p* < 0.001, and body-esteem was negatively associated, *β* = −0.365, *p* < 0.001. The full model accounted for 36.3% of the variance in restrained eating, *R*^2^ = 0.363. The standardized path coefficient plot of the model is provided as Figure S3 in the Supplementary Materials.

Table 7 shows the total, direct, and indirect effects. The total effect was *B* = 0.425 (*β* = 0.152), and the direct effect was reduced to *B* = 0.129 (*β* = 0.046) after including the mediators. The total indirect effect was 0.296 (95% *CI* [0.239, 0.357]), accounting for 69.7% of the total effect. Detailed decomposition of the indirect effect into specific paths and their corresponding percentages is provided in Table 7. The largest indirect association was carried by body-esteem (61.5%), whereas the serial path through self-objectification and body-esteem was small (3.0%). The completely standardized indirect effect for the body-esteem path was in the medium range (*β* = 0.093), while effects for the self-objectification path (*β* = 0.008) and the serial path (*β* = 0.005) were small.

#### 3.3.4. Generalized Others of Perceived Sociocultural Pressure and Restrained Eating

Table 8 presents the serial mediation regression results for generalized others’ sociocultural pressure. After controlling for sex and BMI, generalized others’ pressure was positively associated with self-objectification, *β* = 0.167, *p* < 0.001, *R*^2^ = 0.056. In the second step, with additional controls for self-objectification, generalized others’ pressure was negatively associated with body-esteem, *β* = −0.320, *p* < 0.001; self-objectification also showed a negative association, *β* = −0.088, *p* < 0.001, *R*^2^ = 0.310. In the final step, when all variables were included, generalized others’ pressure was positively associated with restrained eating, *β* = 0.094, *p* < 0.001; self-objectification was positively associated, *β* = 0.063, *p* < 0.01, and body-esteem was negatively associated, *β* = −0.343, *p* < 0.001. The full model accounted for 36.9% of the variance in restrained eating, *R*^2^ = 0.369. The standardized path coefficient plot of the model is provided as Figure S4 in the Supplementary Materials.

Table 9 shows the total, direct, and indirect effects. The total effect was *B* = 0.538 (*β* = 0.220), and the direct effect was reduced to *B* = 0.231 (*β* = 0.094) after including the mediators. The total indirect effect was 0.307 (95% *CI* [0.254, 0.364]), accounting for 57.1% of the total effect. Detailed decomposition of the indirect effect into specific paths and their corresponding percentages is provided in Table 9. The largest indirect association was carried by body-esteem (50.0%), whereas the serial path through self-objectification and body-esteem was small (2.3%). The completely standardized indirect effect for the body-esteem path was in the medium range (*β* = 0.110), while effects for the self-objectification path (*β* = 0.011) and the serial path (*β* = 0.005) were small.

#### 3.3.5. Media of Perceived Sociocultural Pressure and Restrained Eating

Table 10 presents the serial mediation regression results for media sociocultural pressure. After controlling for sex and BMI, media pressure was positively associated with self-objectification, *β* = 0.184, *p* < 0.001, *R*^2^ = 0.061. In the second step, with additional controls for self-objectification, media pressure was negatively associated with body-esteem, *β* = −0.291, *p* < 0.001; self-objectification also showed a negative association, *β* = −0.089, *p* < 0.001, *R*^2^ = 0.292. In the final step, when all variables were included, media pressure was positively associated with restrained eating, *β* = 0.126, *p* < 0.001; self-objectification was positively associated, *β* = 0.057, *p* < 0.05; and body-esteem was negatively associated, *β* = −0.336, *p* < 0.001. The full model accounted for 37.5% of the variance in restrained eating, *R*^2^ = 0.375. The standardized path coefficient plot of the model is provided as Figure S5 in the Supplementary Materials.

Table 11 shows the total, direct, and indirect effects. The total effect was *B* = 0.558 (*β* = 0.239), and the direct effect was reduced to *B* = 0.293 (*β* = 0.126) after including the mediators. The total indirect effect was 0.266 (95% *CI* [0.218, 0.317]), accounting for 47.6% of the total effect. Detailed decomposition of the indirect effect into specific paths and their corresponding percentages is provided in Table 11. The largest indirect association was carried by body-esteem (40.9%), whereas the serial pathway through self-objectification and body-esteem was small (2.3%). The completely standardized indirect effect for the body-esteem path was in the medium range (*β* = 0.098), while effects for the self-objectification path (*β* = 0.011) and the serial path (*β* = 0.006) were small.

### 3.4. Exploratory Multi-Group Analysis by Gender

On the basis of the primary serial multiple mediation model across the full sample, with BMI included as a covariate, supplementary subgroup analyses stratified by gender were further conducted. Previous body image studies have confirmed stable gender differences in responses to sociocultural appearance pressure, self-objectification, body-esteem, and restrained eating among young adults. Accordingly, this section aims to explore whether the sequential associative pathway proposed in the core theoretical model presents heterogeneous characteristics between female and male college students.

The current subgroup examination focuses exclusively on the serial mediation model constructed with the total score of perceived sociocultural pressure (PSP), which corresponds to the core research hypothesis of this study. Analyses for the four subdimensions of PSP were only performed within the full-sample main results to distinguish disparate sources of appearance-related pressure. Given the exploratory attribute of this supplementary analysis, and to avoid redundant tables and overly lengthy content, subgroup mediation examinations for the four PSP subdimensions are not included in the present manuscript. Relevant gender comparisons across separate pressure dimensions can be explored in dedicated follow-up research. Detailed subgroup statistical results are presented in the following sections.

#### 3.4.1. Gender Differences in Descriptive Statistics

Descriptive statistics and sex differences are reported in Table S1. Female participants scored significantly higher than male participants on perceived sociocultural pressure, media of perceived sociocultural pressure, self-objectification, and restrained eating, while reporting notably lower body-esteem and parent of perceived sociocultural pressure (all *p*s < 0.05). Although significant group differences in age and BMI were detected statistically, the effect magnitudes were trivial given the large sample size.

#### 3.4.2. Pearson Correlation Matrices Separated by Gender

Gender disparities in bivariate correlation patterns are illustrated in Table S2. Distinct correlational differences were observed between subgroups. Self-objectification was significantly positively correlated with restrained eating among females (r = 0.180, *p* < 0.01), whereas this association was non-significant for males (r = 0.008, *p* = 0.841). Similarly, the negative correlation between self-objectification and body-esteem was statistically significant in the female group (r = −0.180, *p* < 0.01) but absent among males (r = −0.030, *p* = 0.472). Furthermore, the association between total perceived sociocultural pressure and restrained eating was substantially stronger for females (r = 0.400) than in males (r = 0.242).

#### 3.4.3. Subgroup Serial Mediation Results

The full serial multiple mediation model was estimated separately for female and male subgroups with BMI controlled as a covariate; total, direct, and indirect effects are summarized in Table S3. For female participants, the total standardized indirect effect was significant (*B* = 0.131, 95% *CI* [0.108, 0.156]), with all three specific indirect paths reaching statistical significance. Among these paths, the indirect pathway solely via body-esteem (Ind2) contributed the largest effect magnitude (*B* = 0.115, 95% *CI* [0.093, 0.139]), corresponding to a moderate-to-large effect size.

By contrast, only the indirect path through body-esteem (Ind2) remained significant among male participants (*B* = 0.069, 95% *CI* [0.044, 0.098]). The two pathways involving self-objectification (Ind1 and the serial Ind3 path) were non-significant, with their 95% CIs spanning zero. Additionally, the direct effect of PSP on restrained eating remained significant in the female sample (*B* = 0.123, 95% *CI* [0.083, 0.164]), whereas the direct association was non-significant for males (*B* = 0.044, 95% *CI* [−0.012, 0.101]).

#### 3.4.4. Moderated Serial Mediation Analysis (Gender as Moderator)

To formally test whether the indirect effects differed by sex, a moderated mediation analysis was conducted using PROCESS Model 59, with perceived sociocultural pressure (PSP) as the independent variable, self-objectification (OB) and body-esteem (BE) as sequential mediators, restrained eating (RS) as the dependent variable, gender set as the moderator, and BMI included as a covariate in all regression equations. Bias-corrected bootstrap analyses with 5000 resamples were used to test conditional indirect effects and moderated mediation indices.

The model predicting self-objectification was significant (*R*^2^ = 0.063, *p* <0.001), while the PSP × gender interaction was non-significant (*p* = 0.795), meaning gender could not moderate the PSP–OB linkage. The model for body-esteem fitted well (*R*^2^ = 0.336, *p* <0.001), with a significant PSP × gender interaction (*B* = −0.145, *p* <0.001). The negative predictive effect of PSP on BE was stronger for females (Effect = −0.423, *p* <0.001) than for males (Effect = −0.278, *p* <0.001). The overall model for restrained eating was significant (*R^2 =^* 0.376, *p* <0.001). Only the PSP × gender interaction reached significance (*B* = 0.069, *p* = 0.0481), whereas Ob × gender and BE × gender interactions were non-significant. The direct effect of PSP on RS was highly significant for females (Effect = 0.123, *p* <0.001) and marginally significant for males (Effect = 0.0538, *p* = 0.0505), and OB significantly predicted RS only in the female group.

The index of moderated mediation was not significant for the path through self-objectification (PSP → OB → RS; index = 0.011, 95% *CI* [−0.003, 0.026]), indicating that the indirect association through self-objectification did not differ reliably between female and male participants. However, the index was significant for the path through body-esteem (PSP → BE → RS; index = 0.036, 95% *CI* [0.002, 0.069]), confirming that the indirect association through body-esteem was significantly stronger in female participants (conditional indirect effect = 0.109, 95% *CI* [0.087, 0.132]) than in male participants (conditional indirect effect = 0.073, 95% *CI* [0.049, 0.101]).

To sum up, gender acted as a significant moderator for the direct PSP–RS pathway and the indirect pathway mediated by body-esteem. Although body-esteem exerted a meaningful mediating effect for both males and females, this mediating function was markedly stronger among female participants. By comparison, no reliable gender difference was identified for the indirect path via self-objectification. These moderated mediation outcomes are highly consistent with the gender heterogeneity revealed in the subgroup serial mediation analyses.

## 4. Discussion

The primary aim of this study was to examine the correlational pathway linking perceived sociocultural pressure to restrained eating, as well as the serial mediating roles of self-objectification and body-esteem within this pathway. The current results verified that elevated perceived sociocultural appearance pressure correlates with higher levels of restrained eating among Chinese college students, which is consistent with previous empirical evidence [31]. Within the prevailing thin-ideal aesthetic culture, external sociocultural pressure drives individuals to pursue slim body standards intensely [74], and weight-loss-related perceived pressure has been widely proven to associate positively with restrained eating behaviors [75]. Moreover, the results showed that perceived sociocultural pressure, whether from family, friends, generalized others, or the media, was associated with restrained eating. This indicates that restrained eating may stem from multiple reasons [76].

Sociocultural pressure from the family may exert a pervasive and cumulative influence on restrained eating through subtle yet persistent mechanisms embedded in daily life experiences. According to social learning theory [77], eating behaviors can also be learned through observation and imitation. The pursuit of “thinness” (e.g., dieting, weight commentary) by family members (especially parents) becomes an object of observation for children, leading to imitation of restrained eating [78]. In addition, social norm theory [79] can explain the influence of friends and other people’s pressures to be thin on restrained eating. This theory holds that individuals tend to adjust their behaviors to conform to perceived norms within their social groups. When a friend group regards “thinness” as the ideal body type and openly promotes it, individuals will follow this norm by adopting restrictive diets (such as dieting and calorie counting) to avoid being excluded or to gain social acceptance. Crandall [80] found that restrained eating among female undergraduates exhibits obvious social contagion and interpersonal weight criticism substantially increases individual dieting frequency. From the media perspective, social comparison theory illustrates that mass media constantly broadcasts idealized thin body images of celebrities and models, which readily induces upward appearance comparison among audiences [81,82]. This exposure triggers upward social comparisons, which are particularly detrimental when individuals internalize perceived discrepancies between their own bodies and these narrowly defined media standards, and can further lead to restrained eating [83].

This study focused on the impact of sociocultural pressure from different perspectives, combining social learning theory, social norms theory, and social comparison theory to further identify the potential mechanisms between perceived sociocultural pressure and restrained eating. Consistent with a previous study [2], this study also demonstrated that self-objectification is a mediating variable for perceived sociocultural pressure and restrained eating. Rooted in Objectification Theory, self-objectification is recognized as a critical psychological risk factor for disordered eating and restrained eating [42,43,84]. Chronic outward appearance monitoring induced by self-objectification easily triggers body shame and excessive appearance evaluation; compared with individuals with low self-objectification, those scoring higher are more likely to engage in restrained eating to improve physical appearance and attractiveness [85,86,87]. Notably, these dominant appearance standards are largely disseminated by media and reinforced by family and peer feedback [88], which jointly constitute key antecedents of self-objectification.

This study also verified the independent mediating role of body-esteem in the association between sociocultural pressure and restrained eating. Consistent with the core viewpoint of the Tripartite Influence Model [30,89], sociocultural appearance norms are core predictors of body-related self-evaluation. Adolescents experiencing heightened body dissatisfaction are prone to restrictive dieting to alleviate negative body perceptions [90,91]. It is necessary to clarify that although body-esteem is closely related to body dissatisfaction and both reflect physical self-evaluation [69], body-esteem specifically represents positive subjective satisfaction toward one’s physique rather than negative dissatisfaction [56], which aligns with our definition of body-esteem in the Introduction section and effectively avoids conceptual confusion. The negative correlation between sociocultural pressure and body-esteem, as well as the negative association between body-esteem and restrained eating, fully supports Hypothesis 3. Practically, enhancing positive body-esteem can serve as a promising intervention target to relieve restrained eating risks among young adults.

Most existing studies based on Objectification Theory only test the single mediating role of self-objectification or separately discuss body-esteem as an outcome variable. The present study innovatively constructs a complete serial chain, sociocultural pressure → self-objectification → body-esteem → restrained eating, which clarifies the sequential statistical associative order of two core body image variables, rather than treating them as parallel influencing factors. This result can partly explain inconsistent conclusions in the previous literature: some scholars reported weak direct effects of self-objectification on restrained eating, because body-esteem acts as an indispensable intermediate buffer variable that cannot be omitted from the pathway.

Furthermore, most Western studies under the Tripartite Influence Model focus heavily on media and peer pressure, while this research confirms that family pressure has equally strong predictive effects in Chinese college student samples. The collectivistic cultural background of China makes family aesthetic values exert more lasting implicit influence on youth body cognition compared with Western individualistic contexts, which reflects the localized adaptation of the Tripartite Influence Model in Eastern cultural settings. Combined with the biopsychosocial framework proposed [92], this research supplements environmental social factors and internal cognitive processes in the Chinese youth population, expanding the external applicability of the classic body image theoretical system.

Most importantly, self-objectification and body-esteem were confirmed to function as sequential serial mediators in the target pathway, echoing the correlation between the two psychological constructs reported in existing research [60]. From a theoretical perspective, this serial chain enriches the application scope of Objectification Theory within Chinese youth samples, demonstrating that body-esteem acts as a vital protective buffer between self-objectification and restrained eating. Body-esteem is a core positive dimension of body image, consisting of positive affective attitudes toward one’s own physical features, which is conceptually distinct from body dissatisfaction [93,94]. As proposed by Objectification Theory [42], persistent self-objectification focuses attention exclusively on outward appearance evaluation, which tends to reduce positive body-esteem and aggravate body dissatisfaction simultaneously. The complete external-to-internal sequential path constructed in this study perfectly matches the biopsychosocial framework of body image proposed by Rodgers et al. [92], which systematically integrates environmental sociocultural factors and internal cognitive-emotional processes and further validates the rationality of our serial mediation model design instead of a parallel mediation structure.

Beyond full-sample findings, exploratory multi-group analyses clearly identified significant gender heterogeneity across the hypothesized pathway, which aligns with the gender disparity characteristics of body image and appearance pressure summarized in recent empirical research by Nerini et al. [62,63]. Specifically, female college students reported significantly higher levels of perceived sociocultural pressure, self-objectification, and restrained eating, accompanied by lower body-esteem, relative to male participants. The correlation patterns also presented prominent gender differences: the associations between self-objectification and body-esteem/restrained eating were statistically significant only in the female subgroup. Serial mediation results demonstrated that the complete sequential pathway (perceived sociocultural pressure → self-objectification → body-esteem → restrained eating) held significant exclusively for females. For males, only the indirect path mediated by body-esteem remained effective, while the self-objectification-linked chain failed to reach significance. Moderated mediation analysis further confirmed that gender significantly moderated the direct path from sociocultural pressure to restrained eating and the indirect pathway via body-esteem. Although body-esteem acted as a reliable protective mediator for both genders, its buffering effect was markedly stronger among females, whereas the indirect effect of self-objectification showed no meaningful gender difference.

The gendered pattern can be reasonably interpreted based on Objectification Theory: women in Chinese society face far more frequent external appearance scrutiny from daily social environments, which readily induces trait self-objectification; sustained appearance monitoring sequentially erodes positive body-esteem and ultimately correlates with increased restrained eating behaviors. In contrast, mainstream social norms impose far less appearance evaluation pressure on men, which explains why the self-objectification-mediated pathway becomes non-significant for the male subgroup. Nevertheless, body-esteem maintains stable protective effects across both genders, which is highly consistent with our overall full-sample mediation results. Consistent with the research conclusions of Nerini et al. [62], males are mainly affected by internal physical competence evaluation rather than outward appearance objectification, which fundamentally leads to gender divergence in the psychological mechanism of restrained eating. In line with Objectification Theory, Chinese young women endure more pervasive societal appearance evaluation, which easily triggers self-objectification and sequentially damages body-esteem to promote restrained eating. In contrast, males are less affected by appearance-related social norms, resulting in a non-functional self-objectification pathway. Still, body-esteem acts as a consistent protective mediator across genders, consistent with our main full-sample results.

Notably, these gender analyses are post hoc exploratory supplements.

Given the cross-sectional design, causal conclusions cannot be confirmed, and future research with balanced gender samples may further verify these gender-specific mechanisms.

## 5. Implications and Limitations

At the theoretical level, the present study yields two key advancements. First, this study clarifies the direct association between multi-source perceived sociocultural pressure and restrained eating among Chinese college students, expanding the applicability of the acceptance model of restrained eating to Eastern young adult samples. Second, this study validates the serial mediating pathway of self-objectification and body-esteem, which realizes the organic integration of the Tripartite Influence Model and Objectification Theory. Combined with the biopsychosocial body image framework proposed by Rodgers et al. [92], this research clarifies the external–internal sequential mechanism from environmental appearance pressure to maladaptive eating behaviors and clarifies the sequential rather than parallel predictive effect of self-objectification and body-esteem, supplementing empirical evidence for body image mechanism research in Chinese collectivist cultural contexts.

On a practical level, current findings provide hierarchical, gender-differentiated targeted intervention strategies to mitigate college students’ restrained eating behaviors. Universally, body-esteem acts as a stable protective factor against restrained eating [27], so enhancing positive body-esteem should be regarded as a core intervention target. Specifically, body function identification therapy can be adopted to guide young adults to focus on physical functionality rather than superficial appearance, which effectively lowers self-objectification levels [95] and further alleviates restrained eating tendencies [96]. Beyond body-esteem cultivation, targeted coping strategies toward sociocultural appearance pressure are indispensable. Psychological interventions can help college students rebuild positive self-talk and appearance acceptance mechanisms, replace negative body-related criticism, break the restriction–gluttony vicious cycle, and reduce the risk of clinical eating pathology [97]. Additionally, improving critical media literacy helps individuals deconstruct internalized thin-ideal norms; relevant stakeholders can advocate body-diverse media content and reduce the dissemination of ultra-thin aesthetic images to weaken the adverse effects of sociocultural beauty standards.

Moreover, verified gender heterogeneity enables precise gender-specific intervention design. For female college students who are vulnerable to external appearance evaluation, intervention programs should prioritize reducing appearance self-monitoring and self-objectification, alongside systematic body-esteem education. For male college students, the self-objectification-related pathway is non-significant, so intervention plans can focus on improving overall physical competence evaluation instead of appearance-oriented aesthetic guidance. Furthermore, joint efforts from multiple environments are required: families are suggested to reduce inappropriate weight-related comments, peer communities advocate diversified body aesthetics, and university mental health courses integrate positive body image education to prevent unhealthy restrained eating on campus.

This study has some limitations. First, the cross-sectional designs prevents us from confirming the temporal sequence and causal directions among variables. Although we tested the theoretically proposed serial mediation pathway from perceived sociocultural pressure to restrained eating, alternative model specifications including reverse causal paths are statistically plausible. Longitudinal multi-wave data are needed in future studies to verify the directional relationships and compare competing models. Second, our sample only covers full-time Chinese college students aged 18–24, restricting the generalization of conclusions to adolescents, working adults, or non-Asian populations. Subsequent research can expand sampling ranges to test whether the serial mediation and gender moderation effects remain stable across age groups and cultural backgrounds. Third, this study only examined gender as a moderator; future work can further explore potential moderators such as body mass index, self-compassion, and family socioeconomic status to build a more comprehensive moderated mechanism model. Fourth, the measurement of perceived sociocultural pressure in this study focused only on the pressure to be thin and could not fully represent sociocultural pressure. Future research should focus on the influence of different aspects of sociocultural pressure (e.g., social desirability, social adaptiveness, and thin-ideal internalization) on restrained eating to further expand the prevention and intervention of restrained eating. Meanwhile, all data relied on self-report questionnaires, which may bring response bias. Future research could combine objective behavioral indicators to collect more robust evidence.

## 6. Conclusions

Perceived sociocultural pressure and self-objectification are significantly positively correlated with restrained eating among Chinese college students, while body-esteem is a protective factor that alleviates restrained eating tendencies. This study validates the serial associative pathway linking sociocultural pressure to restrained eating via sequential self-objectification and body-esteem, which integrates the Tripartite Influence Model and Objectification Theory and complements localized body image evidence from Eastern cultural contexts. Notably, prominent gender heterogeneity exists in this serial mechanism: the full serial pathway is statistically significant only for females, whereas only the body-esteem-mediated pathway holds valid for males. The current findings emphasize that targeted, gender-differentiated interventions focusing on lowering multi-source appearance pressure, reducing maladaptive self-objectification, and cultivating positive body-esteem can effectively reduce the prevalence of restrained eating among young adults [98]. Considering the cross-sectional design of this study, future longitudinal research is required to verify the temporal order of the examined variables.

## Figures and Tables

**Table 1:** Correlations, means, and standard deviations of study variables.

Variable	M	SD	Correlations									
			1. Sex	2. BMI	3. RS	4. PSP	5. P_F	6. P_P	7. P_O	8. P_M	9. BE	10. OB
1. Sex	1.67	0.47	1									
2. BMI	21.51	3.12	−0.129 **	1								
3. RS	12.12	5.62	0.261 **	0.369 **	1							
4. PSP	20.11	7.11	0.074 **	0.204 **	0.350 **	1						
5. P_F	4.90	2.02	0.060 *	0.140 **	0.275 **	0.832 **	1					
6. P_P	4.13	2.01	−0.049 *	0.232 **	0.223 **	0.742 **	0.574 **	1				
7. P_O	5.69	2.30	0.053 *	0.163 **	0.296 **	0.849 **	0.621 **	0.465 **	1			
8. P_M	5.38	2.41	0.161 **	0.137 **	0.334 **	0.825 **	0.548 **	0.432 **	0.646 **	1		
9. BE	15.86	6.94	−0.139 **	−0.397 **	−0.522 **	−0.461 **	−0.369 **	−0.343 **	−0.403 **	−0.381 **	1	
10. OB	0.80	11.52	0.170 **	−0.039	0.161 **	0.186 **	0.136 **	0.093 **	0.168 **	0.199 **	−0.154 **	1

Note: BMI = body mass index; M = mean; SD = standard deviation; RS = restrained eating; PSP = perceived sociocultural pressure; P_F = friends of perceived sociocultural pressure; P_P = parents of perceived sociocultural pressure; P_O = generalized others of perceived sociocultural pressure; P_M = media of perceived sociocultural pressure; BE = body-esteem; OB = self-objectification. * *p* < 0.05, ** *p* < 0.01.

**Table 2:** Serial-mediated regression analysis of perceived sociocultural pressure on restrained eating.

	Regression Equation	Overall Fit Index			Significance of Regression Coefficient	
Outcome Variable	Predictive Variable	R	*R* ^2^	F	*β*	t
OB	Sex	0.250	0.062	39.279 ***	0.148	6.356 ***
	BMI				−0.059	−2.460 *
	PSP				0.187	7.927 ***
BE	Sex	0.580	0.337	224.350 ***	−0.144	−7.228 ***
	BMI				−0.344	−17.162 ***
	OB				−0.074	−3.722 ***
	PSP				−0.366	−18.074 ***
RS	Sex	0.611	0.373	210.165 ***	0.228	11.654 ***
	BMI				0.246	11.671 ***
	OB				0.059	3.018 **
	BE				−0.328	−14.170 ***
	PSP				0.121	5.622 ***

Note: BMI = body mass index; M = mean; SD = standard deviation; RS = restrained eating; PSP = perceived sociocultural pressure; BE = body-esteem; OB = self-objectification. * *p* < 0.05, ** *p* < 0.01, *** *p* < 0.001.

**Table 3:** Total, direct, and indirect effects in the serial mediation model.

Effect	Path	*B*	Boot SE	Boot 95% *CI*	*β*	Boot 95% *CI* (*β*)	% of Total Effect
Total effect	X → Y	0.202	0.016	[0.171, 0.234]	0.256	—	—
Direct effects	X → Y	0.095	0.017	[0.062, 0.129]	0.121	—	—
Total mediation effect		0.107	0.010	[0.089, 0.126]	0.136	[0.113, 0.160]	52.90%
Indirect 1	X → M1 → Y	0.009	0.003	[0.003, 0.016]	0.011	[0.004, 0.020]	4.30%
Indirect 2	X → M2 → Y	0.095	0.009	[0.078, 0.113]	0.120	[0.099, 0.143]	46.90%
Indirect 3	X → M1 → M2 → Y	0.004	0.001	[0.002, 0.006]	0.005	[0.002, 0.008]	1.80%

Note. *B* = unstandardized coefficient; *β* = completely standardized coefficient; Boot SE = bootstrap standard error; Boot 95% *CI* = bias-corrected bootstrap 95% confidence interval based on 5000 samples. % of Total Effect = B_indirect/B_total × 100%.

**Table 4:** Serial-mediated regression analysis of friends of perceived sociocultural pressure for restrained eating.

	Regression Equation	Overall Fit Index			Significance of Regression Coefficient	
Outcome Variable	Predictive Variable	R	*R* ^2^	F	*β*	t
OB	Sex	0.214	0.046	28.432 ***	0.157	6.691 ***
	BMI				−0.037	0.086
	P_F				0.132	5.590 ***
BE	Sex	0.544	0.296	185.997 ***	−0.153	7.496 ***
	BMI				−0.380	18.667 ***
	OB				−0.103	5.031 ***
	P_F				−0.293	14.356 ***
RS	Sex	0.61	0.37	206.20 ***	0.228	11.626 ***
	BMI				0.250	11.863 ***
	OB				0.067	3.411 ***
	BE				−0.347	−15.417 ***
	P_F				0.089	4.355 ***

Note: BMI = body mass index; M = mean; SD = standard deviation; RS = restrained eating; P_F = friends of perceived sociocultural pressure; BE = body-esteem; OB = self-objectification. *** *p* < 0.001.

**Table 5:** Total, direct, and indirect effects in the serial mediation model.

Effect	Path	*B*	Boot SE	Boot 95% *CI*	*β*	Boot 95% *CI* (*β*)	% of Total Effect
Total effect	X → Y	0.568	0.057	[0.456, 0.680]	0.204	—	—
Direct effects	X → Y	0.248	0.057	[0.136, 0.359]	0.089	—	—
Total mediation effect		0.321	0.031	[0.265, 0.383]	0.115	[0.095, 0.137]	56.40%
Indirect 1	X → M1 → Y	0.024	0.009	[0.008, 0.043]	0.009	[0.003, 0.015]	4.30%
Indirect 2	X → M2 → Y	0.283	0.029	[0.230, 0.343]	0.102	[0.083, 0.123]	49.80%
Indirect 3	X → M1 → M2 → Y	0.013	0.004	[0.006, 0.021]	0.005	[0.002, 0.008]	2.30%

Note. *B* = unstandardized coefficient; *β* = completely standardized coefficient; Boot SE = bootstrap standard error; Boot 95% *CI* = bias-corrected bootstrap 95% confidence interval based on 5000 samples. % of Total Effect = B_indirect/B_total × 100%.

**Table 6:** Serial-mediated regression analysis of family of perceived sociocultural pressure regarding restrained eating.

	Regression Equation	Overall Fit Index			Significance of Regression Coefficient	
Outcome Variable	Predictive Variable	R	*R* ^2^	F	*β*	t
OB	Sex	0.200	0.041	25.120 ***	0.170	7.229 ***
	BMI				−0.043	−1.794
	P_P				0.111	4.647 ***
BE	Sex	0.525	0.275	167.986 ***	−0.180	8.668 ***
	BMI				−0.365	17.414 ***
	OB				−0.113	−5.498 ***
	P_P				−0.256	12.226 ***
RS	Sex	0.603	0.363	201.844 ***	0.232	11.722 ***
	BMI				0.246	11.543 ***
	OB				0.071	3.619 ***
	BE				−0.365	−16.376 ***
	P_P				0.046	0.247 *

Note: BMI = body mass index; M = mean; SD = standard deviation; RS = restrained eating; P_P = family of perceived sociocultural pressure; BE = body-esteem; OB = self-objectification. * *p* < 0.05, *** *p* < 0.001.

**Table 7:** Total, direct, and indirect effects in the serial mediation model.

Effect	Path	*B*	Boot SE	Boot 95% *CI*	*β*	Boot 95% *CI* (*β*)	% of Total Effect
Total effect	X → Y	0.425	0.059	[0.309, 0.541]	0.152	—	—
Direct effects	X → Y	0.129	0.057	[0.016, 0.241]	0.046	—	—
Total mediation effect		0.296	0.03	[0.239, 0.357]	0.106	[0.086, 0.128]	69.70%
Indirect 1	X → M1 → Y	0.022	0.008	[0.008, 0.040]	0.008	[0.003, 0.014]	5.20%
Indirect 2	X → M2 → Y	0.261	0.029	[0.207, 0.320]	0.093	[0.074, 0.114]	61.50%
Indirect 3	X → M1 → M2 → Y	0.013	0.004	[0.006, 0.021]	0.005	[0.002, 0.008]	3.00%

Note. *B* = unstandardized coefficient; *β* = completely standardized coefficient; Boot SE = bootstrap standard error; Boot 95% *CI* = bias-corrected bootstrap 95% confidence interval based on 5000 samples. % of Total Effect = B_indirect/B_total × 100%.

**Table 8:** Serial-mediated regression analysis of generalized others of perceived sociocultural pressure on restrained eating.

	Regression Equation	Overall Fit Index			Significance of Regression Coefficient	
Outcome Variable	Predictive Variable	R	*R* ^2^	F	*β*	t
OB	Sex	0.237	0.056	35.112 ***	0.155	6.638 ***
	BMI				−0.047	−1.970 *
	P_O				0.167	7.120 ***
BE	Sex	0.557	0.310	199.103 ***	−0.155	−7.657 ***
	BMI				−0.368	−18.211 ***
	OB				−0.088	−4.332 ***
	P_O				−0.320	−15.725 ***
RS	Sex	0.607	0.369	206.686 ***	0.230	11.699 ***
	BMI				0.249	11.813 ***
	OB				0.063	3.229 **
	BE				−0.343	−15.072 ***
	P_O				0.094	4.529 ***

Note: BMI = body mass index; M = mean; SD = standard deviation; RS = restrained eating; P_O = generalized others of perceived sociocultural pressure; BE = body-esteem; OB = self-objectification. * *p* < 0.05, ** *p* < 0.01, *** *p* < 0.001.

**Table 9:** Total, direct, and indirect effects in the serial mediation model.

Effect	Path	*B*	Boot SE	Boot 95% *CI*	*β*	Boot 95% *CI* (*β*)	% of Total Effect
Total effect	X → Y	0.538	0.05	[0.439, 0.636]	0.22	—	—
Direct effects	X → Y	0.231	0.051	[0.131, 0.330]	0.094	—	—
Total mediation effect		0.307	0.028	[0.254, 0.364]	0.125	[0.104, 0.149]	57.10%
Indirect 1	X → M1 → Y	0.026	0.01	[0.009, 0.047]	0.011	[0.003, 0.019]	4.80%
Indirect 2	X → M2 → Y	0.269	0.027	[0.218, 0.324]	0.11	[0.089, 0.132]	50.00%
Indirect 3	X → M1 → M2 → Y	0.012	0.004	[0.006, 0.020]	0.005	[0.002, 0.008]	2.30%

Note. *B* = unstandardized coefficient; *β* = completely standardized coefficient; Boot SE = bootstrap standard error; Boot 95% *CI* = bias-corrected bootstrap 95% confidence interval based on 5000 samples. % of Total Effect = B_indirect/B_total × 100%.

**Table 10:** Serial-mediated regression analysis of media of perceived sociocultural pressure on restrained eating.

	Regression Equation	Overall Fit Index			Significance of Regression Coefficient	
Outcome Variable	Predictive Variable	R	*R* ^2^	F	*β*	t
OB	Sex	0.248	0.061	38.588 ***	0.134	5.678 ***
	BMI				−0.047	−2.007 *
	P_M				0.184	7.799 ***
BE	Sex	0.540	0.292	182.197 ***	−0.126	−6.075 ***
	BMI				−0.377	−18.427 ***
	OB				−0.089	−4.305 ***
	P_M				−0.291	−13.933 ***
RS	Sex	0.612	0.375	211.789 ***	0.217	11.014 ***
	BMI				0.248	11.811 ***
	OB				0.057	2.928 *
	BE				−0.336	−15.051 ***
	P_M				0.126	6.066 ***

Note: BMI = body mass index; M = mean; SD = standard deviation; RS = restrained eating; P_M = media of perceived sociocultural pressure; BE = body-esteem; OB = self-objectification. * *p* < 0.05, *** *p* < 0.001.

**Table 11:** Total, direct, and indirect effects in the serial mediation model.

Effect	Path	*B*	Boot SE	Boot 95% *CI*	*β*	Boot 95% *CI* (*β*)	% of Total Effect
Total effect	X → Y	0.558	0.048	[0.464, 0.652]	0.239	—	—
Direct effects	X → Y	0.293	0.048	[0.198, 0.387]	0.126	—	—
Total mediation effect		0.266	0.026	[0.218, 0.317]	0.114	[0.094, 0.136]	47.60%
Indirect 1	X → M1 → Y	0.025	0.009	[0.008, 0.044]	0.011	[0.003, 0.019]	4.40%
Indirect 2	X → M2 → Y	0.228	0.024	[0.183, 0.276]	0.098	[0.079, 0.118]	40.90%
Indirect 3	X → M1 → M2 → Y	0.013	0.004	[0.006, 0.021]	0.006	[0.003, 0.009]	2.30%

Note. *B* = unstandardized coefficient; *β* = completely standardized coefficient; Boot SE = bootstrap standard error; Boot 95% *CI* = bias-corrected bootstrap 95% confidence interval based on 5000 samples. % of Total Effect = B_indirect/B_total × 100%.

## Data Availability

The data presented in this study are available on request from the corresponding author due to they are derived from the Behavioral Brain Research Project of Chinese Personality (BBP).

## Supplementary Materials

The following supporting information can be downloaded at: https://www.mdpi.com/article/10.3390/nu18132142/s1, Figure S1: Serial mediation model testing self-objectification and body esteem as mediators between perceived sociocultural pressure and restrained eating. Figure S2: Serial mediation model testing self-objectification and body esteem as mediators between friends of perceived sociocultural pressure and restrained eating. Figure S3: Serial mediation model testing self-objectification and body esteem as mediators between family of perceived sociocultural pressure and restrained eating. Figure S4: Serial mediation model testing self-objectification and body esteem as mediators between generalized others of perceived sociocultural pressure and restrained eating. Figure S5: Serial mediation model testing self-objectification and body esteem as mediators between media of perceived sociocultural pressure and restrained eating. Table S1: Descriptive Statistics and Sex Differences. Table S2: Bivariate Correlations by Sex. Table S3: Total, Direct, and Indirect Effects by Sex.
