# Supplementary material for: Perceived Sociocultural Pressure and Restrained Eating Among Chinese College Students: The Serial Mediating Roles of Self-Objectification and Body-Esteem
Source: Nutrients. 2026 Jul 2;18(13):2142. doi: 10.3390/nu18132142 (PMC13363584; doi:10.3390/nu18132142)
Supplement: Supplementary file 1 [file nutrients-18-02142-s001.zip › Figure_S5.pdf]

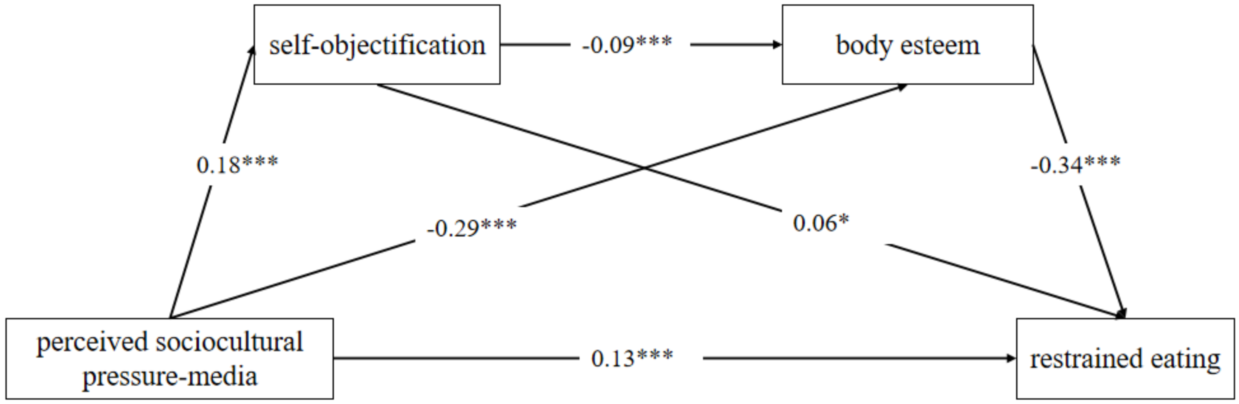

Figure S5. Serial mediation model testing self-objectification and body esteem as mediators between media of perceived sociocultural pressure and restrained eating. Standardized coefficients, *$p < .05$, ***$p < .001$.
