# Supplementary material for: Perceived Sociocultural Pressure and Restrained Eating Among Chinese College Students: The Serial Mediating Roles of Self-Objectification and Body-Esteem
Source: Nutrients. 2026 Jul 2;18(13):2142. doi: 10.3390/nu18132142 (PMC13363584; doi:10.3390/nu18132142)
Supplement: Supplementary file 1 [file nutrients-18-02142-s001.zip › nutrients-4387984-supplementary tables.pdf]

TableS1. Descriptive Statistics and Sex Differences

Variable	Female (n= 1195)		Male (n= 579)		t	p
	M	SD	M	SD		
Age	19.22	0.99	19.41	1.26	3.57	< .001
BMI	21.23	2.78	22.09	3.66	5.46	< .001
PSP	20.48	6.93	19.35	7.42	3.14	0.002
P_F	4.98	2.00	4.73	2.05	2.51	0.012
P_P	4.06	1.99	4.28	2.04	2.09	0.037
P_O	5.78	2.28	5.52	2.32	2.22	0.027
P_M	5.65	2.38	4.83	2.39	6.86	< .001
OB	2.16	11.51	−2.01	11.03	7.25	< .001
BE	15.19	6.94	17.25	6.75	5.92	< .001
RS	13.14	5.29	10.01	5.70	11.40	< .001

Note: BMI = body mass index; M = mean; SD = standard deviation; RS = restrained eating; PSP = perceived sociocultural pressure; P_F = friends of perceived sociocultural pressure; P_P = family of perceived sociocultural pressure; P_O = generalized others of perceived sociocultural pressure; P_M = media of perceived sociocultural pressure; BE = body-esteem; OB = self-objectification. t-tests were based on equal variances not assumed where Levene's test was significant. All p-values are two-tailed.

TableS2. Bivariate Correlations by Sex

Variable	1	2	3	4	5	6	7	8	9
1. BMI	—	.184**	.520**	.142**	.186**	.144**	.151**	−.330**	−.078
2. PSP	.241**	—	.242**	.866**	.803**	.853**	.850**	−.381**	.182**
3. RS	.355**	.400**	—	.231**	.179**	.205**	.202**	−.431**	0.008
4. P_F	.156**	.814**	.289**	—	.682**	.663**	.608**	−.332**	.149**
5. P_P	.258**	.720**	.280**	.527**	—	.518**	.553**	−.339**	.083*
6. P_O	.192**	.847**	.340**	.598**	.443**	—	.667**	−.336**	.195**
7. P_M	.170**	.814**	.361**	.516**	.393**	.637**	—	−.282**	.179**
8. BE	−.488**	−.495**	−.547**	−.381**	−.360**	−.431**	−.407**	—	−.030
9. OB	0.019	.174**	.180**	.117**	.112**	.146**	.176**	−.180**	—

Note: Female participants (n = 1195) below the diagonal; male participants (n = 579) above the diagonal. BMI = body mass index; M = mean; SD = standard deviation; RS = restrained eating; PSP = perceived sociocultural pressure; P_F = friends of perceived sociocultural pressure; P_P = family of perceived sociocultural pressure; P_O = generalized others of perceived sociocultural pressure; P_M = media of perceived sociocultural pressure; BE = body-esteem; OB = self-objectification. *p < .05, **p < .01(two-tailed).

Table S3. Total, Direct, and Indirect Effects by Sex

Effect	Pathway	Female (n= 1195)			Male (n= 579)		
		B	95% CI	β	B	95% CI	β
Total effect	PSP → RS	0.255	[0.215, 0.294]	0.333	0.117	[0.063, 0.170]	0.152
Direct effect	PSP → RS	0.123	[0.083, 0.164]	0.162	0.044	[−0.012, 0.101]	0.058
Total indirect		0.131	[0.108, 0.156]	0.172	0.072	[0.046, 0.101]	0.094
Ind1	PSP → OB → RS	0.011	[0.004, 0.020]	0.014	0.004	[−0.008, 0.015]	0.005
Ind2	PSP → BE → RS	0.115	[0.093, 0.139]	0.150	0.069	[0.044, 0.098]	0.090
Ind3	PSP → OB → BE → RS	0.006	[0.003, 0.010]	0.008	0.000	[−0.004, 0.003]	−0.001

Note. B = unstandardized coefficient; β = completely standardized coefficient; 95% CI = bias-corrected bootstrap 95% confidence interval based on 5,000 samples. Covariate: BMI. PSP = perceived sociocultural pressure; RS = restrained eating; OB = self-objectification.; BE = body-esteem.
